# A systems biology approach towards the identification of candidate therapeutic genes and potential biomarkers for Parkinson’s disease

**DOI:** 10.1371/journal.pone.0220995

**Published:** 2019-09-05

**Authors:** Meena Kishore Sakharkar, Sarinder Kaur Kashmir Singh, Karthic Rajamanickam, Musthafa Mohamed Essa, Jian Yang, Saravana Babu Chidambaram

**Affiliations:** 1 Drug Discovery and Development Research Group, College of Pharmacy and Nutrition, University of Saskatchewan, Saskatoon, Canada; 2 Institute of Biological Sciences, Faculty of Science, University of Malaya, Kuala Lumpur, Malaysia; 3 Sultan Qaboos University, Muscat, Oman; 4 Department of Pharmacology, JSS College of Pharmacy, JSSAHER, Karnataka, India; Hokkaido Daigaku, JAPAN

## Abstract

Parkinson’s disease (PD) is an irreversible and incurable multigenic neurodegenerative disorder. It involves progressive loss of mid brain dopaminergic neurons in the substantia nigra pars compacta (SN). We compared brain gene expression profiles with those from the peripheral blood cells of a separate sample of PD patients to identify disease-associated genes. Here, we demonstrate the use of gene expression profiling of brain and blood for detecting valid targets and identifying early PD biomarkers. Implementing this systematic approach, we discovered putative PD risk genes in brain, delineated biological processes and molecular functions that may be particularly disrupted in PD and also identified several putative PD biomarkers in blood. 20 of the differentially expressed genes in SN were also found to be differentially expressed in the blood. Further application of this methodology to other brain regions and neurological disorders should facilitate the discovery of highly reliable and reproducible candidate risk genes and biomarkers for PD. The identification of valid peripheral biomarkers for PD may ultimately facilitate early identification, intervention, and prevention efforts as well.

## Introduction

Parkinson’s disease (PD) affects about 1% of the population above the age of 65 and is a progressive and disabling neurodegenerative disease. Its prevalence increases with age. It affects 41 people per 100,000 in the age group of 30–40 years old to over 1900 per 100,000 in people over 80 years of age [1]. Mechanistically, it involves a progressive loss of mid brain dopaminergic neurons in the substantia nigra pars compacta (SN). PD is clinically characterized by motor (tremors, rigidity, gait disturbance, impaired handwriting, grip force and speech deficits), non-motor (Mild cognitive impairment (PD-MCI) and dementia (PDD) symptoms. Reports show that by the time the clinical motor symptoms are expressed, more than 60% of dopaminergic neurons degenerate. As of today, there is no cure for PD and pharmacological manipulations provide only symptomatic relief. Currently, treatments with carbidopa-levodopa, dopamine agonists, monoamine oxidase B (MAO-B)-inhibitors and catechol-O-methyltransferase (COMT) inhibitors aim at improving brain dopamine levels. Hence, both the decrease of symptoms and the potential for slowing disease progression can have major impacts on the treatment and prognosis of this chronic disease. Here, it is important to mention that as of today, the diagnosis of PD is clinical and there is no diagnostically conclusive test for PD. In the early stages of PD, when the spectrum of clinical signs required to accurately confirm the diagnosis is partial, diagnosing PD can be a real challenge. To this end, besides identifying treatment options for palliative care, it is imperative to identify biomarkers to detect early PD and investigate how these biomarkers can be used in drug development and clinical trials. Because of this, it is important to mention that biopsies from several peripheral tissues like skin, olfactory and gastrointestinal (GI) tissues have been explored for the identification of biomarkers in PD. Data analysis is hampered by the diversity of the methods used e.g. choice of biopsy sites, tissue processing, staining techniques and analyses of the findings.

In parallel, peripheral blood biomarkers have been of interest for early detection, diagnosis and risk management for several pathological conditions including PD. The identification of biomarkers in blood can also help in treatment planning and prediction of therapeutic response. In comparison to traditional tissue biopsies, which are feasible in PD patients (unless they are post-mortem), peripheral blood sampling for biomarker identification is non-invasive. It also allows for easy repeated sampling to analyze temporal patterns, disease progression and the effects of treatment.

Also, compared to the traditional single gene based analyses, microarrays can simultaneously analyze the roles of multiple genes in a disorder. This provides for the identification of additional novel risk factors besides the known key factors implicated in the progression of the disease. This is specifically imperative for identifying risk factors for a complex disorder like PD, which is thought to have a multifactorial mutigenic etiology, in which many genes and environmental factors interplay [2]. Data on differentially expressed genes in the SN region of PD patients’ brains and healthy controls can provide useful information to identify the molecular mechanisms implicated in disease etiology and potential therapeutic targets. Investigating mechanistic and potentially causal interpretations of the differentially expressed gene (DEG) data from blood and SN region of the brain followed and mapping into contextual framework in biochemical pathways involved in the disease can be used to evaluate and propose potential drug-targets and biomarkers for diagnostics. Here, we performed a pairwise comparison of the gene expression pattern between peripheral blood samples and the lateral and medial regions of the SN tissue samples of PD patients to their respective healthy control samples. The results of our investigation are presented.

## Methodology

### Microarray data

The microarray datasets for GDS2519 and GDS3128 were downloaded from Gene Expression Omnibus (GEO). These datasets are based on the platform of Affymetrix Human Gene 1.0 ST Array. The dataset GDS3128 contains data for lateral and medial region in substantia nigra (SN) from post-mortem brain samples obtained from 47 individuals with sporadic Parkinson's disease (PD) and healthy controls. The dataset GDS2519 contains whole blood data of 50 patients predominantly with early-stage Parkinson’s disease and 22 healthy controls [3].

### Data preprocessing

The original CEL data was imported into R and the Affy package was used for background correction and normalization. After the microarray data normalizing and standardization, we identified the list of differentially expressed genes (DEGs) between GDS2519 and healthy controls and GDS3128 and healthy controls, respectively using Limma from R [4]. P values < 0.05 and log_2_fold change >1.0 was chosen as cut-off standards for GDS3128 and P values < 0.05 and log_2_fold change >0.5 were chosen as cut-off standard for GDS2519. DEGs that are common to these two datasets at these cut-offs were identified. The top 10 upregulated and downregulated genes in brain and blood are listed in Table 1 and Table 2.

**Table 1:** Top 10 downregulated and up-regulated genes in PD patient brain (SN).

Gene name	Adj.P.Val	P.Value	logFC	Gene description
SYT1	0.000298	2.96E-06	-2.9551989	synaptotagmin 1
TH	0.0074116	5.62E-04	-2.8951615	tyrosine hydroxylase
DDC	0.0004568	6.22E-06	-2.7808595	dopa decarboxylase
DLK1	0.0001051	1.79E-07	-2.6298578	delta like non-canonical Notch ligand 1
RGS4	0.0006494	1.13E-05	-2.5894884	regulator of G-protein signaling 4
VSNL1	0.0098521	9.13E-04	-2.5006865	visinin like 1
PEG10	0.0002226	1.55E-06	-2.4748904	paternally expressed 10
SLC18A2	0.0066241	4.63E-04	-2.4109851	solute carrier family 18 member A2
FGF13	0.0000745	7.36E-08	-2.3413631	fibroblast growth factor 13
NMNAT2	0.0002644	2.23E-06	-2.3019162	nicotinamide nucleotide adenylyltransferase 2
CEBPD	0.0002696	2.34E-06	1.6360053	CCAAT/enhancer binding protein delta
VSIG4	0.0028116	1.21E-04	1.798724	V-set and immunoglobulin domain containing 4
XIST	0.0648801	2.24E-02	1.9219418	X inactive specific transcript (non-protein coding)
CD163	0.0038071	1.94E-04	2.0190042	CD163 molecule
SLCO4A1	0.000017	9.87E-10	2.0365554	solute carrier organic anion transporter family member 4A1
IL1RL1	0.0025594	1.04E-04	2.0657307	interleukin 1 receptor like 1
AZGP1	0.000437	5.53E-06	2.1063662	alpha-2-glycoprotein 1, zinc-binding
CD163	0.0013422	3.61E-05	2.1642491	CD163 molecule
MAFF	0.0001641	7.81E-07	2.2522527	MAF bZIP transcription factor F
NPTX2	0.0003113	3.19E-06	2.5077172	neuronal pentraxin 2

**Table 2:** Top 10 downregulated and up-regulated genes in PD patient peripheral blood.

Gene name	Adj.P.Val	P.Value	logFC	Gene Description
DLG1	0.689	0.0003131	-1.26172	discs large MAGUK scaffold protein 1
PURG	0.741	0.0034109	-1.0817409	purine rich element binding protein G
XIST	0.819	0.0310748	-1.0538732	X inactive specific transcript (non-protein coding)
NUDT4P1///NUDT4	0.689	0.0002757	-1.0395957	nudix hydrolase 4 pseudogene 1///nudix hydrolase 4
HLA-DQB1	0.689	0.00143	-0.9912498	major histocompatibility complex, class II, DQ beta 1
PDE6D	0.689	0.0005137	-0.9853906	phosphodiesterase 6D
ABCD2	0.809	0.006745	-0.9833886	ATP binding cassette subfamily D member 2
ATP8A1	0.689	0.0020289	-0.974955	ATPase phospholipid transporting 8A1
ZFAND1	0.741	0.0033109	-0.9602558	zinc finger AN1-type containing 1
IFI27	0.689	0.0020649	-0.9581476	interferon alpha inducible protein 27
KRT20	0.809	0.0060482	0.9746298	keratin 20
LINC00302	0.741	0.0034608	0.9893661	long intergenic non-protein coding RNA 302
MAGEA2B///MAGEA2	0.809	0.0077282	1.0005549	MAGE family member A2B///MAGE family member A2
TARP	0.809	0.006297	1.0164369	TCR gamma alternate reading frame protein
EMP1	0.741	0.0033636	1.0327375	epithelial membrane protein 1
PCDH7	0.809	0.0078986	1.0426983	protocadherin 7
GLRA3	0.689	0.0013667	1.0490169	glycine receptor alpha 3
PRKAG2	0.689	0.0005499	1.148714	protein kinase AMP-activated non-catalytic subunit gamma 2
DDX3Y	0.809	0.022047	1.2936699	DEAD-box helicase 3, Y-linked
RPS4Y1	0.809	0.0155187	1.4363633	ribosomal protein S4, Y-linked 1

### Functional annotation and pathway analysis of DEGs

Gene Ontology (GO) summarizes information on the functional knowledge of gene products by describing the cellular and organismal processes. In order to make a comprehensive evaluation of relevant pathway or biological procedures in PD, GO analyses were carried out using the software FunRich ver. 3.0 (http://www.funrich.org) on DEGs for both the datasets and pathway enrichment analyses on DEGs for both the datasets were carried out using GeneAlaCart. GeneAlaCart is a web search engine that generates a file of annotations associated with a supplied list of genes (https://genealacart.genecards.org/). Key super-pathways for the DEGs were also identified using annotations supplied by GeneAlaCart.

### Analysis of protein interaction networks

PPI networks were created using the BioGrid database. The proteins in the PPI network act as “nodes” where each protein–protein interaction pair is represented by an undirected link and the degree of a node represents the number of interactions of a protein. The most connected nodes (high degree) are called “hubs”. These hubs were chosen for additional analysis. The network for the common genes was visualized by Cytoscape (ver 3.3.0) [5].

## Results

**Fig 1:**
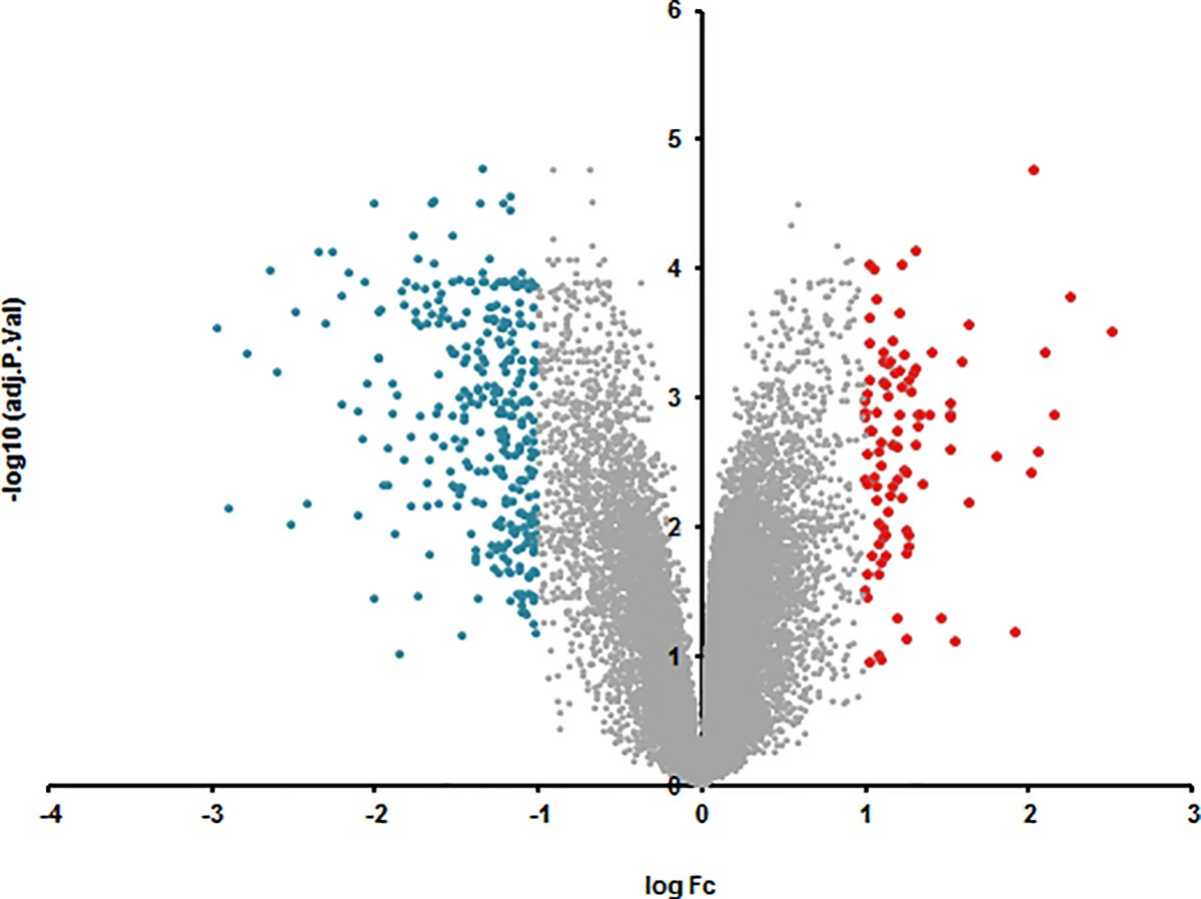
Volcano plot of the retrieved microarray data for brain, plotting the negative log10 of the adjusted P-value against the log2 of the fold change (FC).

**Fig 2:**
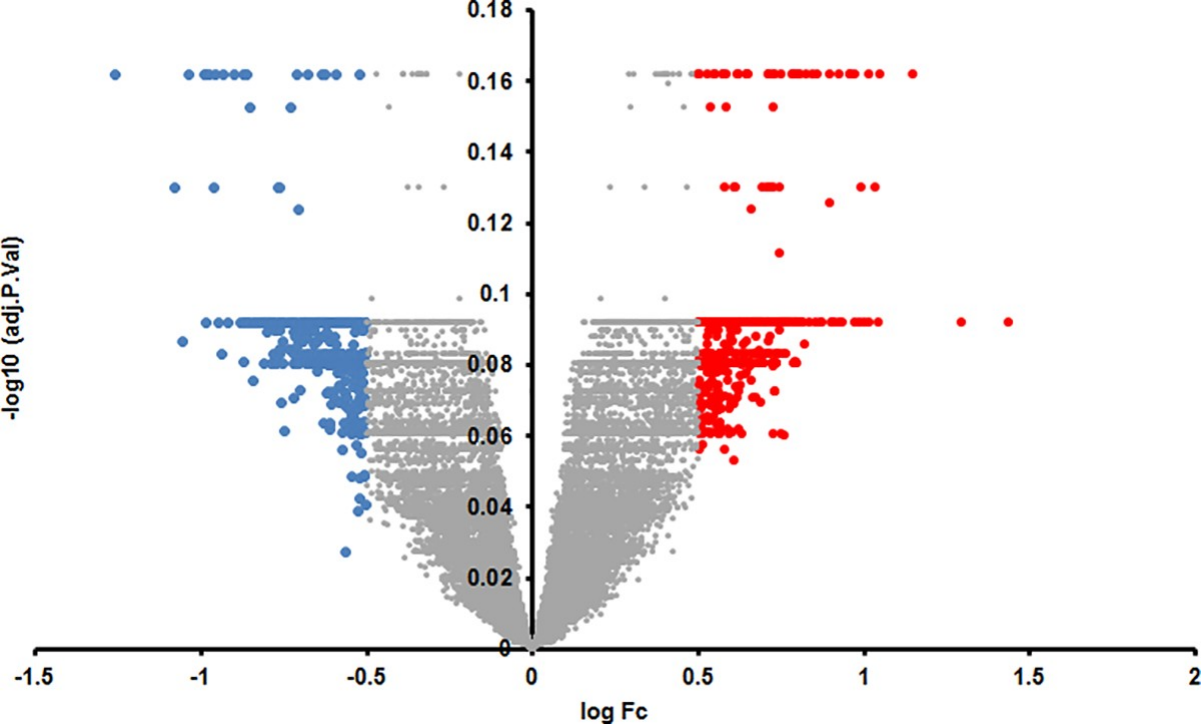
Volcano plot of the retrieved microarray data for blood, plotting the negative log10 of the adjusted P-value against the log2 of the fold change (FC).

We identified 316 genes which were downregulated and 98 genes that were upregulated at least log_2_ one fold in the SN. We identified 4 genes which were downregulated at least log_2_ one fold and 8 genes that were upregulated at least log_2_ one fold in parkinsonian whole blood samples (Set A). This number of genes in blood was very low for target identification and hence we lowered our cut-off to log_2_ 0.5 fold change in expression in blood samples and identified 426 downregulated and 517 upregulated genes (Set B). A distribution of these genes with their log_2_ fold changes is represented in Figs 1 and 2.

**Fig 3:**
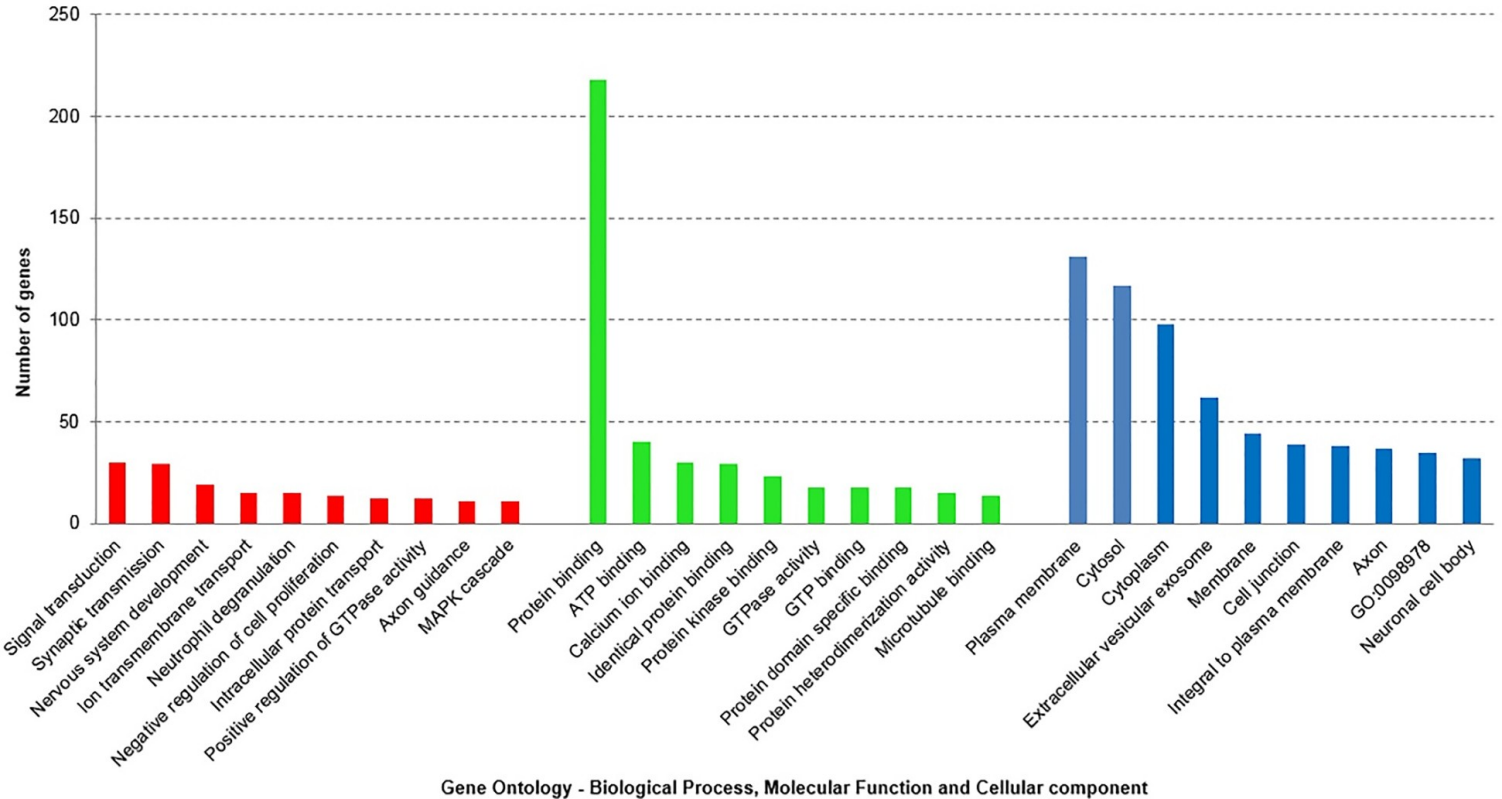
The three organizing principles of GO are biological process, cellular component and molecular function. The histograms represent Gene Ontology (GO) analysis of the differentially expressed (Set A) mRNAs in the brain (SN) PD patients). GO terms according to the three GO organizing principles are represented on X axis. The Y-axis shows number of genes associated with GO term (P < 0.05).

**Fig 4:**
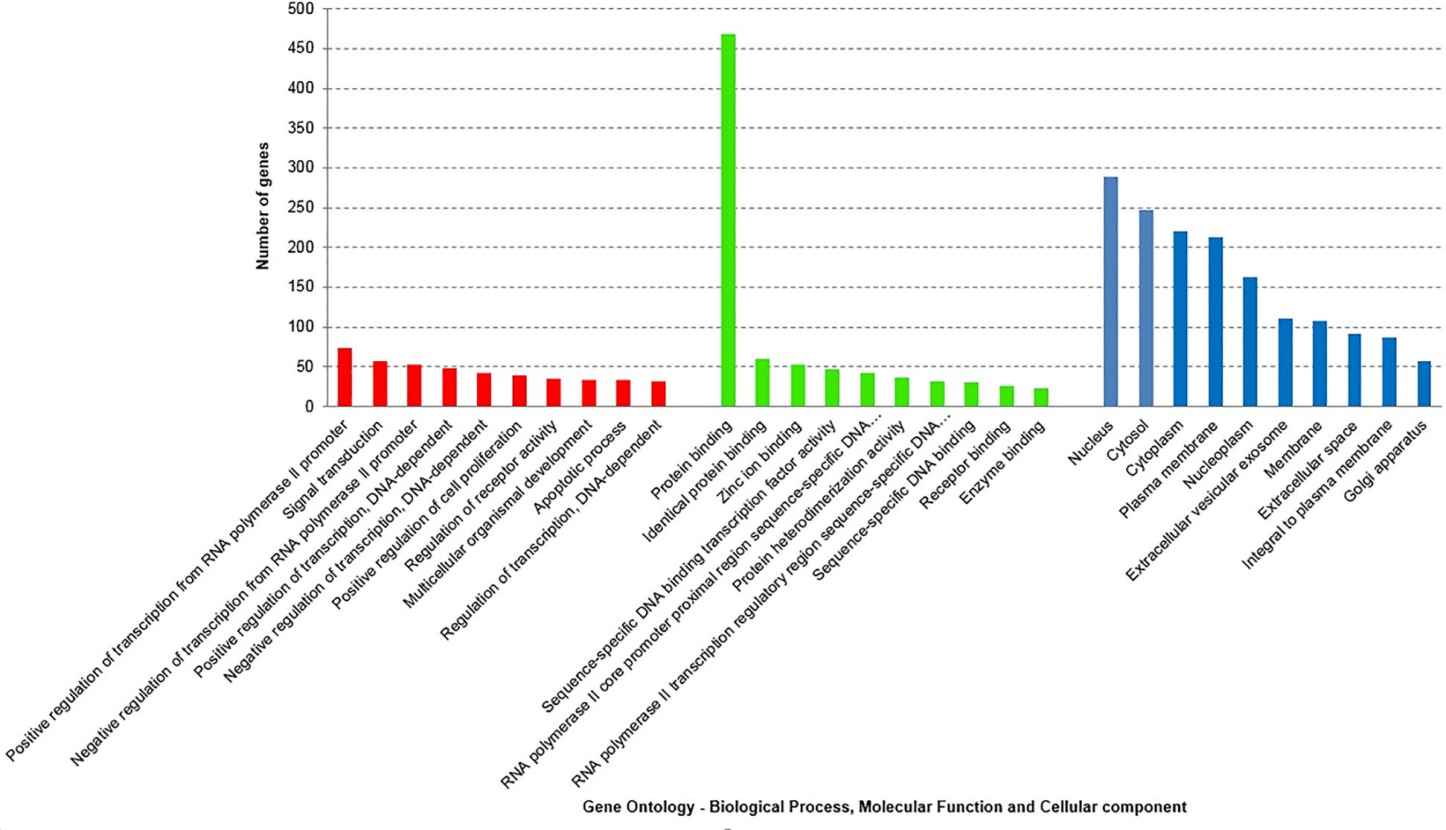
The three organizing principles of GO are biological process, cellular component and molecular function. The histograms represent Gene Ontology (GO) analysis of the differentially expressed (Set A) mRNAs in the blood of PD patients. GO terms according to the three GO organizing principles are represented on X axis. The Y-axis shows number of genes associated with GO term (P < 0.05).

'Gene ontology' (GO) [6] represents a controlled vocabulary to describe gene and gene product attributes in an organism (http://www.geneontology.org). The three organizing principles of GO are molecular function, biological process and cellular component. To acquire information on GO terms associated with the DEG in SN and blood, we subjected the DEG in SN (Set A) i.e. 414(316+98) genes and 943(426+517) DEG in blood (Set B) GO analyses using GeneAlaCart. The top 5 significantly enriched GO terms in biological processes are signal transduction (30), synaptic transmission (29), nervous system development (19), ion transmembrane transport (15), neutrophil degranulation (15) in the brain and positive regulation of transcription from RNA polymerase II promoter (73), signal transduction (57), negative regulation of transcription from RNA polymerase II promoter (53), positive regulation of transcription, DNA—dependent (48) and negative regulation of transcription, DNA—dependent (42) in blood. The top 5 significantly enriched molecular function annotations for genes differentially expressed in SN are protein binding (218), ATP binding (40), calcium ion binding (30), identical protein binding (29), protein kinase binding (23) and protein binding (469), identical protein binding (60), zinc ion binding (52), sequence–specific DNA binding transcription factor activity (42), RNA polymerase II core promoter proximal region sequence-specific DNA (42) in the blood (Figs 3 and 4).

**Fig 5:**
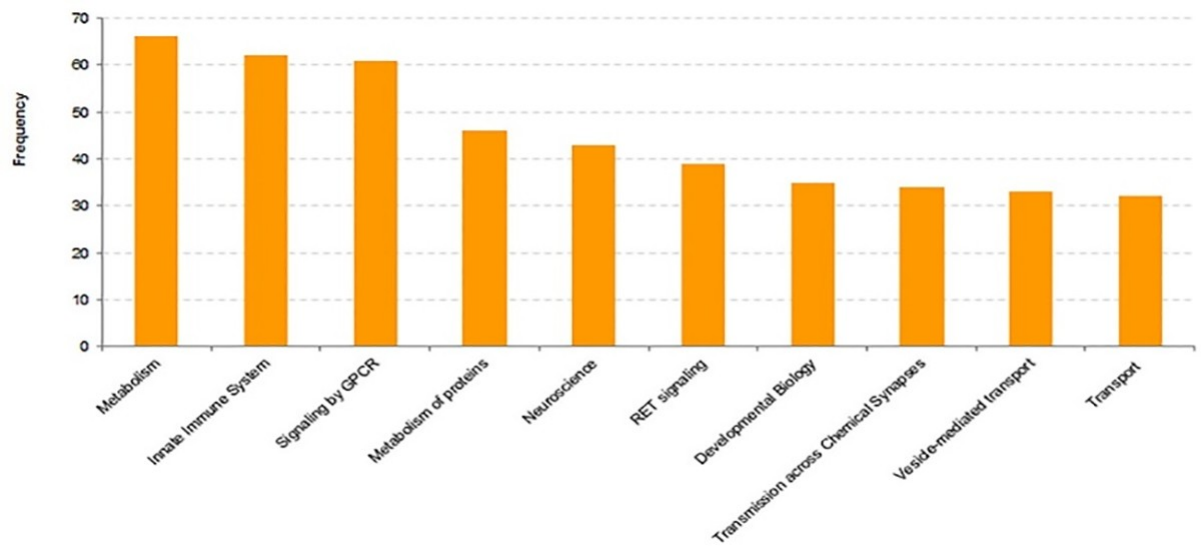
Super pathway analyses of the differentially expressed mRNAs in PD patients (brain). Frequency represents number of genes in the super pathway.

**Fig 6:**
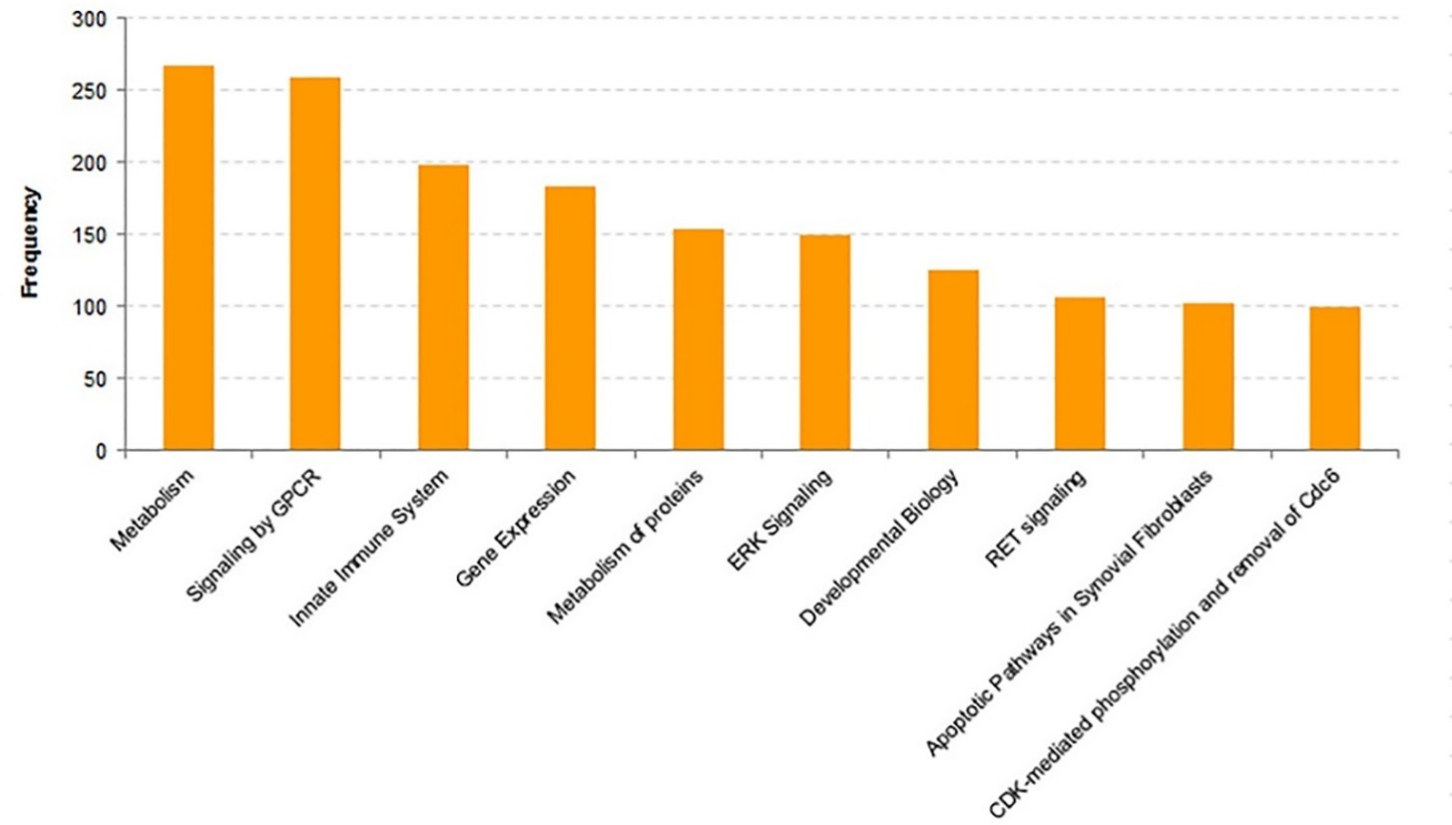
Super pathway analyses of the differentially expressed mRNAs in PD patients (blood). Frequency represents number of genes in the super pathway.

Super-pathway analysis shows that the top 5 DEG category annotations in SN are metabolism (66), Innate Immune System (62), Signaling by GPCR (61), Metabolism of proteins (46), Neuroscience and Metabolism (268), Signaling by GPCR (259), Innate Immune System (199), Gene Expression (184), Metabolism of proteins (154) in the blood (Figs 5 and 6). Here, it must be mentioned that some genes are included in more than one GO terms and more than one super-pathways.

**Fig 7:**
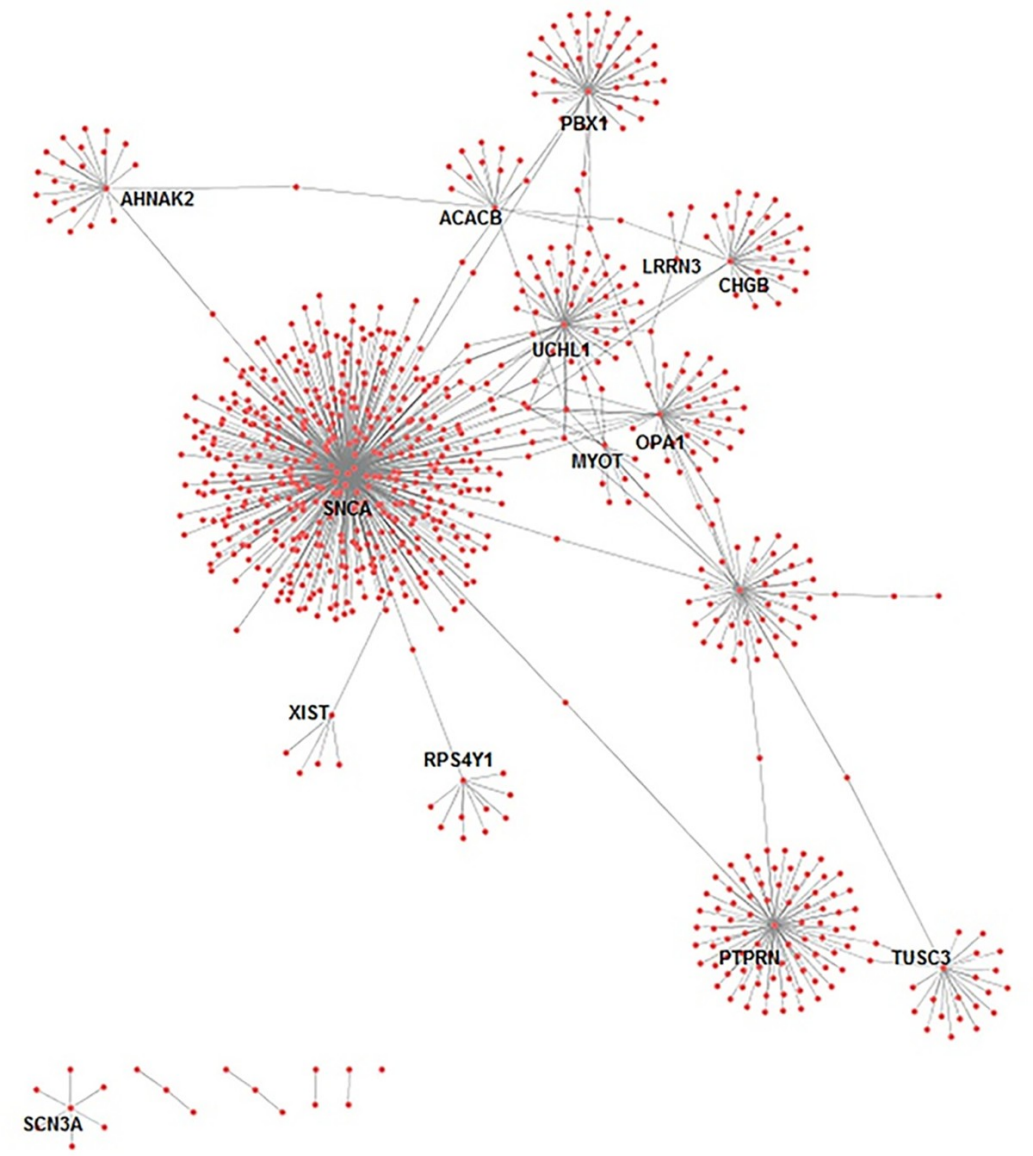
Protein protein interaction network for the genes that are DEG in the brain and the blood.

Interestingly, 20 proteins were found to be differentially expressed in both SN and blood (Table 3). These genes and their function based on GeneCards (https://www.genecards.org/) are elaborated. XIST (X Inactive Specific Transcript—silences one of the X chromosomes during early development), UCHL1 (Ubiquitin C-Terminal Hydrolase L1—is specifically expressed in the neurons and in cells of the diffuse neuroendocrine system) and hydrolyzes a peptide bond at the C-terminal glycine of ubiquitin, TUSC3 (Tumor Suppressor Candidate 3—associated with biological functions such as cellular magnesium uptake, protein glycosylation and embryonic development), SNCA (Synuclein Alpha–Also called PARK1—abundantly expressed in the brain and defects in SNCA have been reported to contribute to the pathogenesis of Parkinson disease), SLC18A2 (Solute Carrier Family 18 Member A2 –involved in the accumulation of cytosolic monoamines into synaptic vesicles and defects are associated with neuropsychiatric disorders), SCN3A (Sodium Voltage-Gated Channel Alpha Subunit 3 –causes generation and propagation of action potentials in neurons and muscle), RPS4Y1 (Ribosomal Protein S4 Y-Linked 1 –involved in protein synthesis), PTPRN (Protein Tyrosine Phosphatase, Receptor Type N–involved in the regulation of a variety of cellular processes), PBX1 (PBX Homeobox 1 –a nuclear protein and transcription factor), ORC5 (Origin Recognition Complex Subunit 5—essential for DNA replication in eukaryotic cells), OPA1 (Mitochondrial Dynamin-Like GTPase–nuclear encoded mitochondrial protein and helps regulate mitochondrial stability and energy output), MYOT (Myotilin—cystoskeletal protein that stabilizes the thin filaments during muscle contraction and defects in this gene are associated with limb-girdle muscular dystrophy and myofibrillar myopathies), MT1H (Metallothionein 1H –involved in metabolic functions), LRRN3 (Leucine Rich Repeat Neuronal 3), KDM5D (Lysine Demethylase 5D - short peptide derived from this protein is a minor histocompatibility antigen which can lead to graft rejection), CHGB (Chromogranin B—encodes a tyrosine-sulfated secretory protein abundant in peptidergic endocrine cells and neurons), CACNB2 (Calcium Voltage-Gated Channel Auxiliary Subunit Beta 2—voltage-dependent calcium channel protein subunit), AHNAK2 (AHNAK Nucleoprotein 2 –the protein plays a role in calcium signaling by associating with calcium channel proteins), ADAM23 (implicated in a variety of biological processes), ACACB(Acetyl-CoA Carboxylase Beta—a biotin-containing enzyme which catalyzes the carboxylation of acetyl-CoA to malonyl-CoA). Further analysis on the interacting partners for the proteins of these 20 genes that are differentially expressed in SN and blood shows that these genes are highly connected and hence can be considered as potential therapeutic targets. (Fig 7)(Table 3)

**Table 3:** List of genes that are DEG in the brain and the blood with a FC of > = 1 and > = 0.5, respectively. TUSC3, SNCA, SLC18A2, SCN3A, PTPRN, PBX1, ORC5, OPA1, LRRN3, CHGB, CACNB2, ADAM23 were found to be downregulated in both PD brain SN and blood. XIST, MYOT, MT1H, ACACB were found to be upregulated in PD brain SN but were found to be down regulated in the blood.

Gene name	Brain	Blood	Degree of connectivity
XIST	1.9219418	-1.0538732	5
UCHL1	-1.4323314	0.7089191	62
TUSC3	-1.2045245	-0.7063718	22
SNCA	-1.5113946	-0.6061047	487
SLC18A2	-2.4109851	-0.5768519	1
SCN3A	-1.1890016	-0.8529165	6
RPS4Y1	-1.8436708	1.4363633	10
PTPRN	-1.172985	-0.684063	85
PBX1	-1.2045702	-0.6837467	49
ORC5	-1.0099365	-0.7632592	57
OPA1	-1.1335631	-0.5198584	44
MYOT	1.5229308	-0.5677766	12
MT1H	1.1073722	-0.5183126	0
LRRN3	-1.1030907	-0.6887866	3
KDM5D	-1.0243291	0.8200079	2
CHGB	-1.9661147	-0.5175298	29
CACNB2	-1.267547	-0.5121574	2
AHNAK2	-1.9766206	0.7734374	22
ADAM23	-1.4506176	-0.510121	1
ACACB	1.1798452	-0.8142551	14

## Discussion

Parkinson’s disease is irreversible as most of the neurons in the part of the brain affected by the disease have already died by the time the patient first experiences motor symptoms. The disease remains incurable due to its elusive mechanisms and there are no disease modifying or neuroprotective treatments for PD therapeutics as of today. Hence, palliative care provides a comprehensive approach for maximizing the quality of life for patients and reducing stress for the duration of the disease [7]. Thus, there is an urgent need to understand the pathophysiology of this multigenic disorder and identify validated targets and biomarkers for early detection of this neurological disease. Pathway analyses of PD shows that it is a complex ailment with links to cancer, diabetes and inflammation [8].

Genomics blood biomarkers have shown tremendous promise for the development of therapeutic options and clinical monitoring and diagnosis of neurodegenerative diseases. Since early intervention could be facilitated by early detection of the PD, several studies on the identification of biomarkers for PD have been conducted. As the signs of molecular and cellular neuropathology of PD start to appear before the start of the distinctive symptoms of PD, early detection of the biomarkers could assist in diagnosis before the irreversible damage occurs. Concurrently, understanding the proteins involved in PD derived from experimental strategies constitutes targets for therapeutic interventions and symptomatic relief.

Here, we have investigated DEG from the SN region of PD brains and blood (against healthy controls) which could be useful for distinguishing PD patients from controls and identifying potential targets for early therapeutics and diagnosis. We also report 20 genes that are differentially expressed in both SN and blood samples (Table 3). There is no consistent pattern in the change of their expression in PD patients when compared to health controls. TUSC3, SNCA, SLC18A2, SCN3A, PTPRN, PBX1, ORC5, OPA1, LRRN3, CHGB, CACNB2 and ADAM23 were found to be downregulated in both the SN region and blood samples of PD patients. The relationship of these genes to pathophysiology of PD is elaborated. Alpha-synuclein (SNCA) is the one of the key therapeutic targets and codes for a protein that forms Lewy bodies. The aggregation of alpha-synuclein and dysfunction of mitochondria (the powerhouse of the cell) are hallmarks of PD. It is expected to be up-regulated in PD patients. Surprisingly, in the present study, SNCA was found to be down-regulated in both the SN and blood of PD patients when compared to healthy controls. Seo et al. reported that low levels of alpha-synuclein regulate neuronal survival through the expression of Bcl-2 family and PI3/Akt kinase pathway [9]. They also reported that at low levels, alpha-synuclein has a protective effect against serum deprivation and oxidative stress, which is mediated by the Akt pathway and overexpression of Bcl-2. Investigations on genes involved in PD have explained on the role of oxidative stress and apoptosis in the degradation of dopaminergic neurons [10]. Interestingly, both in SN region and blood, the levels of Bcl-2/Akt were modulated positively in PD patients. TUSC3 (Tumor Suppressor Candidate 3) is involved in cellular magnesium uptake, protein glycosylation and embryonic development. TUSC3, have been identified on chromosome 8 p-arm. This region has high expression of nervous system related genes and has been identified as a hub for neuropsychiatric development disorders [11]. SLC18A2 is a transporter of monoamines, such as dopamine, norepinephrine, serotonin, and histamines and is reported to be dysfunctional in PD brain [12]. SCN3A codes for a voltage-gated sodium channel and generates and propagates action potential in neurons and muscle and has recently been reported to be associated with epilepsy which is a reported comorbidity of Parkinson's disease (PD) [13]. PTPRN is a tyrosine phosphatase that acts as a signaling molecule and regulates a variety of cellular processes. It is involved in the normal accumulation of the neurotransmitters norepinephrine, dopamine and serotonin in the brain (By similarity). PTPRN is expressed in neurons and PTPRN levels are reported to be depressed in Alzheimer’s disease patients[14]. Since, PTPRN is involved in metabolism and cellular processes, its contribution to metabolic theories of development of PD cannot be ruled out [15]. PBX1 is a nuclear transcriptional factor family protein. It is involved in the regulation of osteogenesis and is required for skeletal patterning and programming. Interestingly, PBX1 transcriptional network has been reported to be involved in controlling dopaminergic neuron development and is impaired in Parkinson's disease [16]. ORC5 initiates DNA replication. To the best of our knowledge, no association between ORC5 and PD has been reported as of today. OPA1 is a nuclear-encoded mitochondrial protein and is involved in the maintenance of mitochondrial genome and is the key protein involved in mitochondria fusion [17, 18]. It is similar to dynamin-related GTPase that regulate the equilibrium between mitochondrial fusion and mitochondrial fission [6, 19–21]. It is also involved in regulating mitochondrial stability and energy output and the sequestering of cytochrome c. Alterations in OPA1 have been reported in PD patients [22]. LRRN3 is a Leucine-rich repeat neuronal protein 3 which is reported to regulate inflammation. Inflammation and T-cell function related genes like LRRN3 and lymphoid enhancer-binding factor 1 (*LEF1*) are upregulated in the younger age group when compared to the old age group [23–27]. This data concurs with the fact that as in the case of the old age group, PD patients are not able to regulate inflammation as effectively as healthy individuals. LRRN3 has recently been reported as a potential biomarker of PD [28]. CHGB encodes a tyrosine-sulfated secretory protein abundant in neurons. This protein may serve as a precursor for regulatory peptides and has a key role in vesicle cargo and exocytosis [29]. It has been reported to be associated with PD [30]. CACNB2 polymorphism is reported to confer susceptibility to schizophrenia and other neurological disorders[31, 32] and possibly regulates neuronal differentiation [33]. However, it is important to mention that, based on the PPI data, the degree of connectivity of three proteins SLC18A2, CACNB2, ADAM23 was found to be below 5. The degree of connectivity is generally a decisive criterion for target prioritisation. However, several FDA approved drugs are available against these targets and are used as therapeutics in in the treatment of PD (Table 4) [34].

**Table 4:** List of genes that are DEG in the brain and the blood and have drugs available (based on the DrugBank database).

Gene Name	Drugbank ID (Drug name)
SCN3A	DB00909 (Zonisamide); DB05232 (Tetrodotoxin); DB06218(Lacosamide); DB00313 (Valproic Acid)
CACNB2	DB00270 (Isradipine); DB00381(Amlodipine); DB00393(Nimodipine); DB00401(Nisoldipine); DB00622 ( Nicardipine); DB00653(Magnesium sulfate); DB00661(Verapamil); DB01023(Felodipine); DB01054 (Nitrendipine); DB01115 (Nifedipine); DB01388 (Mibefradil); DB04855 (Dronedarone); DB06712 (Nilvadipine); DB00421(Spironolactone)
SLC18A2	DB00182 (Amphetamine); DB00206 (Reserpine); DB00368 (Norepinephrine); DB00386 (Rauwolfia serpentina root); DB00865 (Benzphetamine); DB01089 (Deserpidine); DB01363 (Ephedra); DB01364 (Ephedrine); DB01442 (MMDA); DB01454 (Midomafetamine); DB01472 (4-Methoxyamphetamine); DB01576 (Dextroamphetamine); DB01577 (Methamphetamine); DB04844 (Tetrabenazine); DB06706 ( Isometheptene); DB06714(Propylhexedrine)
KDM5D	DB00126 (Vitamin C)
ACACB	DB00121 (Biotin); DB00173 (Adenine); DB02859 (Soraphen A); DB03781 (2-[4-(2,4-Dichlorophenoxy)Phenoxy]Propanoic Acid); DB07870 ((2s)-2-(4-{[3-Chloro-5-(Trifluoromethyl)Pyridin-2-Yl]Oxy}Phenoxy)Propanoic Acid)

Interestingly, three proteins–EGFR, APP and PARK2—which are the interacting partners of the common genes, had a degree of connectivity of more than 5 (Table 5). However, their expression does not change two- or more fold in brain and 0.5 or more fold in blood (as observed from our data analyses). Nonetheless, it is very important to mention that these proteins play significant roles in the brain and mitochondrial biogenesis. Specifically, the EGFR signaling pathway plays a significant role in dopaminergic (DAergic) neuronal death during the process of neuro-apoptosis and therefore can be focused on as a potential target for therapeutic intervention [35]. APP is a cell surface receptor which performs physiological functions on the surface of neurons relevant to neurite growth, neuronal adhesion and axonogenesis. PARK2 (alias—Parkin) regulates Akt signaling by ubiquitinating an adaptor protein that normally binds to the EGFR, a protein called Eps15 [36]. Eps15 is a protein that binds ubiquitin and regulates the internalization and degradation of EGFR. Parkin loss and the resulting decrease in Akt signaling can cause the death of neurons that characterize Parkinson’s disease. Alterations in this gene are reported to cause Parkinson disease and autosomal recessive juvenile Parkinson disease [37]. Thus, it is evident that proteins that are differentially modulated negatively in both the brain and the blood have important functions in neuronal heath, DNA replication, transcription, signaling and apoptosis and mitochondrial function. Mitochondrial dysfunction has been reported to play a key role in the pathogenesis of PD [36, 38]. Thus, the role of interacting proteins as targets is also important [39].

**Table 5:** List of top three interacting partners and their fold change in differential expression.

Gene name	Brain	Blood	Degree of connectivity
APP	-0.1395017	-0.31537349	8
EGFR	0.0222216	-0.12405563	5
PARK2	0.0294328	0.23537164	6

XIST, MYOT, MT1H, ACACB were found to be upregulated in the SN regions of the PD brain but were found to be down regulated in the blood. XIST transcriptionally silences one of the X chromosomes and has been earlier reported to be associated with PD [40]. MYOT (Myotilin) encodes a cystoskeletal protein which stabilises of thin filaments during muscle contraction. Muscle changes are characteristic feature in PD[41]. MT1H is a metallothioneins that has a high content of cysteine residues and binds various heavy metals and is thereby involved in neuroprotection. Their overexpression in PD patients’ SN has been reported [42]. ACACB catalyzes the carboxylation of acetyl-CoA to malonyl-CoA and is involved in the inhibition of fatty acid and glucose oxidation. Dysregulated neurometabolism has been reported to hasten neurodegeneration [43]. Moreover, fatty acids are not only precursors for membrane biosynthesis, lipid signaling molecules, energy storage but neuronal activation brought about by ion channels has also been shown to be regulated by lipids either directly or indirectly [25, 44–47].

UCHL1, KDM5D and AHNAK2 were found to be downregulated in the SN region of a PD brain but were found to be upregulated in the blood of PD patients. UCHL1 encodes a thiol protease and is specifically expressed in the neurons and in cells of the diffuse neuroendocrine system. Mutations in this gene may be associated with Parkinson disease. RPS4Y1 codes for 40S ribosomal protein S4, Y isoform 1, KDM5D is a Histone demethylase that specifically demethylates Lys-4 of histone H3, thereby plays a central role in histone code, AHNAK2 is a nucleoprotein and is involved in calcium signaling by associating with calcium channel proteins.

Proteins encoded by 5 of the identified DEG present in PD brain SN and blood were found to be druggable i.e. they could be subject to modulation by small molecules. The drugs available for these markers are presented in Table 4. Several of these could be potential treatments for PD.

DLG1 shows the highest downregulation (-1.26172) and RPS4Y1 is the most upregulated gene in the blood of PD patients. Discs large 1 (DLG1) is a protein implicated in modular scaffolding which is involved in the assembly of specific multiprotein complexes, including receptors, ion channels and signaling proteins, at specific regions of the plasma membrane [48–50]. It has ubiquitous membranous and cytoplasmic expression as per data in protein atlas database. (https://www.proteinatlas.org/ENSG00000075711-DLG1/tissue). DLG1 and RPS4Y1 have been reported as distinct candidate genes or risk factors for early Parkinson's disease by analysis of gene expression in whole blood [51].

NPTX2 shows the highest upregulation and SYT1 shows the highest downregulation in the SN region of a PD brain. NPTX2 is involved in excitatory synapse formation and causes non-apoptotic cell death of dopaminergic nerve cells. Interestingly, NPTX2 is downregulated in Alzheimer's disease. Though NPTX2 is primarily expressed in the brain, the structure for NPTX2 is unavailable, which is why we could not screen for drugs *in silico*. SYT1 is involved in the regulation of membrane trafficking and is a transmembrane protein. It acts as a Ca2+ sensor in the synaptic vesicles for the release of neurotransmitters [52]. The SYT1 structure is available and has been solved in conjunction with botulinum neurotoxin (4isq). This prompted us to check if the botulinum neurotoxin could be a potential drug for PD. Interestingly, an investigation to evaluate the safety and efficacy of incobotulinumtoxinA (IncoA) injection for the treatment of tremor in PD has recently been reported recently [53]. Moreover, SYT1 is an interesting target since it is expressed at a markedly higher level in the brain (https://www.proteinatlas.org/). This is very promising, as the expression in relevant tissues under normal conditions is a criterion for being the targets of efficacious drugs. As indicated by Kumar et al., genes that play key roles in the physiological processes of complex organisms have tissue-specific patterns of expression and function and such genes can be important contributors to the pathophysiology of the disease and hence, be important targets [54]. This is an excellent example on the use of gene prioritization in picking out the relevant targets from expression data for designing of potential drugs.

In summary, all the DEG in SN brain, blood and/or common genes do not appear to represent a single biologic pathway or process. This reflects on the complexity of this multigenic disorder and our inability to identify the key pathways responsible for this pathophysiological condition. Data on DEG can facilitate early detection, help in diagnosis and facilitate prognosis. However an understanding on mechanistic and functional role is required for evaluating biomarkers for druggability. The current analyses provide a list of therapeutic targets and biomarkers and some knowledge on their importance in PD. Supplementing this information with data from literature on target interactions, validated pathways and druggability will eventually move the promising targets to clinical trials and for therapeutic interventions by drugs.

## Limitations

It must be mentioned that gene expression values for multifactorial diseases such as PD have a wide range and may not simply be present or absent. Thus, caution is advised in using these genes as biomarkers. The clinical heterogeneticity and etiology of the disease and the stage of the disease [55] must be a consideration factor when using them as biomarkers. Moreover, the DEG data from the blood of patients versus healthy populations may help in disease diagnosis and predicting outcomes, but does not give any information on the role of the biomarkers in disease manifestations (causal influences) and disease process (effects).

It must also be kept in view that pre- and postmortem conditions (e.g., coma, hypoxia, hyperpyrexia at the time of death, and long postmortem interval) introduce potential confounds that may influence the quality of tissues. The influence of sampling techniques on the data cannot be ignored. Unfortunately, for most of the patient samples, the data on duration, severity of the disorder, and the treatments the patients underwent prior to tissue donation is not known. The effects of these factors on the respective mRNA expression results are impossible to assess adequately.
